# Learning health systems and evidence ecosystems: a perspective on the future of evidence-based medicine and evidence-based guideline development

**DOI:** 10.1186/s12961-023-01095-2

**Published:** 2024-01-04

**Authors:** D. Rajit, A. Johnson, E. Callander, H. Teede, J. Enticott

**Affiliations:** 1 Monash Centre for Health Research and Implementation, Faculty of Medicine, Nursing, and Health Sciences, Monash University, Level 1, 43-51 Kanooka Grove, Melbourne, VIC 3168 Australia; 2 Monash Partners Academic Health Sciences Centre, Melbourne, VIC Australia; 3 School of Public Health, Faculty of Health, University of Technology Sydney, Sydney, NSW Australia; 4 Monash Health Endocrinology and Diabetes Departments, Melbourne, Australia

**Keywords:** Learning health systems, Evidence ecosystems, Evidence-based medicine, Person-centred care, Evidence-based guidelines

## Abstract

Despite forming the cornerstone of modern clinical practice for decades, implementation of evidence-based medicine at scale remains a crucial challenge for health systems. As a result, there has been a growing need for conceptual models to better contextualise and pragmatize the use of evidence-based medicine, particularly in tandem with patient-centred care. In this commentary, we highlight the emergence of the learning health system as one such model and analyse its potential role in pragmatizing both evidence-based medicine and patient-centred care. We apply the learning health system lens to contextualise the key activity of evidence-based guideline development and implementation, and highlight how current inefficiencies and bottlenecks in the evidence synthesis phase of evidence-based guideline development threaten downstream adherence. Lastly, we introduce the evidence ecosystem as a complementary model to learning health systems, and propose how innovative developments from the evidence ecosystem may be integrated with learning health systems to better enable health impact at speed and scale.

## Introduction

Evidence-based medicine (EBM) is the de facto lens through which modern healthcare is delivered, intending to “integrate individual clinician expertise and patient values with the best external evidence” [1]. First conceived as a suite of methods and tools to systematize the critical appraisal of the medical literature and standardize clinical education and practice [2], EBM’s 40-year track record includes numerous successes in public health, and a historic role in shaping the way an entire generation of clinicians and researchers would deal with research evidence, for example, through the adoption of large bibliographic databases such as MEDLINE [3], and methodologies and tools such as systematic search strategies [4] and systematic review production [5] that now form the backbone of modern evidence synthesis.

However, EBM as a movement has continued to experience its fair share of barriers to system-wide adoption. Historically limited guidance on how best to integrate “patient values” within clinical decision-making and research have led to perceived tensions with person-centred care [13]. Traditionally, hierarchical stances on the “best external evidence” which upholds the randomized controlled trial (RCT) as the gold standard have led to downstream challenges in evidence-based guideline adherence [6], especially in cases of complex morbidity and diverse patient groups [7]. Continued inefficiencies in evidence synthesis [8], exacerbated by ongoing concerns with research waste [9] and integrity [10], also threaten to delay evidence-based responses to health system shocks, as exemplified by the ongoing coronavirus disease 2019 (COVID-19) epidemic [8, 9].

As a result, there has been growing interest in models to better contextualize and integrate the use of EBM in tandem with person-centred care at a whole-of-system level, whilst promoting efficient and timely evidence synthesis. As a solution, we discuss the learning health system (LHS) and how innovative developments from the evidence ecosystem may be integrated to address these challenges, improve the resilience of learning health systems and better enable health impact at speed and scale.

## Learning health systems: towards integrating EBM and person-centred care

**Fig. 1:**
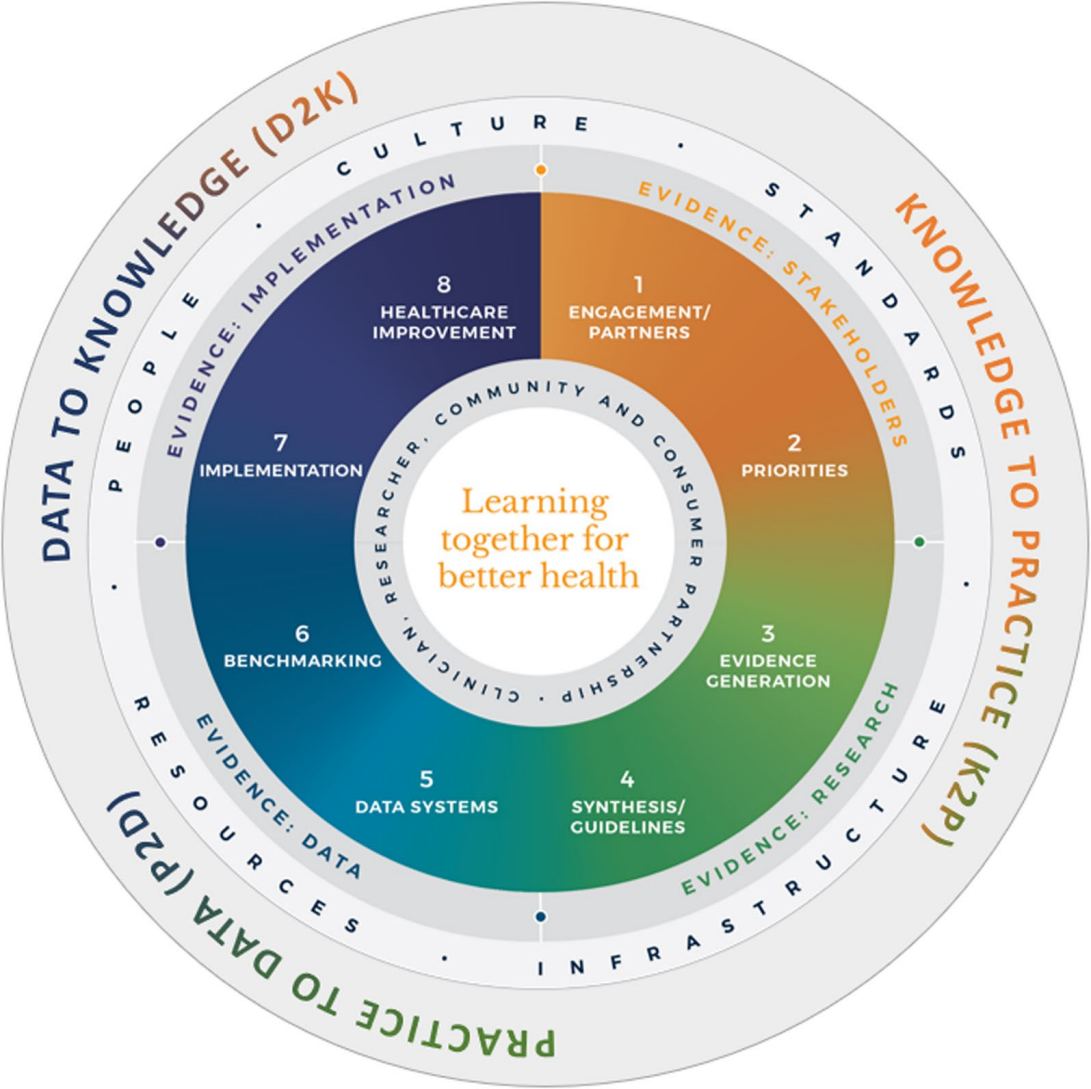
All relevant sources of evidence (Stakeholders, research, data and implementation) within a LHS, contextualised within the learning health cycle (outermost ring)

Learning health systems are models for health systems where people, technology and culture are aligned to enable cyclical, data-driven healthcare improvement, or ‘learning’, at scale [11], with diverse examples across the world now beginning to demonstrate measurable impact [12]. As a meta-framework, or a framework-of-frameworks, the LHS thus contextualises and broadens what constitutes ‘evidence’ in evidence-based clinical practice; alongside recognizing the need to marry the research-based, evidence-based practice of the EBM tradition with contextualized, practice-based evidence stemming from frontline clinician experience and person-centred care, data and lastly, implementation, as illustrated in Fig. 1.

Thus, evidence from stakeholders is generated through deep engagement with relevant stakeholders, including consumers, practitioners, service managers, administrators and other bodies. Delphi approaches [13] and living labs [14] are used to capture stakeholder need, co-design interventions and determine priorities, which are then incorporated into downstream evidence generation such as integrating patient-reported outcome measures (PROMs) and patient-reported experience measures (PREMS) into trial design [15]. Evidence from research, now informed with evidence from stakeholders, is then generated through the traditional tools of EBM such as systematic reviews [16], evidence-based guideline development [17] and the Grading of Recommendations, Assessment, Development and Evaluations (GRADE) [18] framework. Evidence from practice and data is elicited through real-time clinical data such as electronic health records [19], and evidence from implementation is then captured via relevant implementation science frameworks [20] which are applied towards adopting, sustaining and evaluating change. Unmet need surfaced at this point is solicited again as evidence from stakeholders, and the cycle continues as the emergent, learning health cycle (Outer Ring in Fig. 1), with knowledge comprising evidence from stakeholders and research, influencing changes in practice (K2P), changes in practice leading to changes in data (P2D) as captured through evidence from data, and changes in data consequently leading to changes in knowledge (D2K), as captured through evidence from implementation.

As such, the learning health system framework is ideal for addressing problems related to evidence-based guideline development and implementation. Designed to aid in clinical decision-making, evidence-based guidelines (EBGs) are crucial in minimising clinical variation and promoting value-based care. However, the historical implementation and adherence of guidelines has been highly fraught with challenges, with a recent meta-review [6] indicating a potential for lack of practical relevance of guidelines when produced. Firstly for patients, for example, due to language and literacy barriers; and secondly to the health professional, due to factors such as the perceived lack of credible evidence within the guidelines [6]. Insufficient sociotechnical infrastructure to support guideline implementation is also a barrier, such as a lack of clear leadership or implementation champion, alongside resource constraints preventing practitioners from keeping up to date with the guidelines [6].

In response, the learning health system can be used to contextualise the process of EBG development and implementation (Table 1), addressing issues of relevance at multiple levels. Firstly, further upstream during the first three phases of EBG development which includes establishing the scope of the EBG (Phase 1), undertaking the systematic review of evidence (Phase 2), and formulating evidence based guidance (Phase 3), by explicity positioning evidence from stakeholders such as prioritised clinical questions and outcomes as key evidence to consider during EBG scoping. This ensures alignment early in the process, helping ensure that downstream systematic review production and evidence synthesis during phase 2 addresses the questions that matter to stakeholder; and recommendations formulated during phase 3 are evidence-based, patient-centred and once informed by frameworks such as the GRADE Feasibility, Cost Acceptability, and Ethnicity (FACE) approach [21], ready for dissemination and implementation.

**Table 1:** LHS evidence quadrants mapped against the various phases of EBG development

LHS evidence quadrant	EBG development phase	Example activity
Evidence from stakeholders	Phase 1: Establish GDG and scope	The development of the 2018 International PCOS Guidelines involved extensive engagement with 3500 stakeholders in partnerships, surveys and workshops to set priorities and rank outcomes of importance for guideline inclusion [25]
Evidence from research	Phase 2: Systematic review of evidence Phase 3: Formulate guidance	The development of the 2018 International PCOS Guidelines featured the production of 19 systematic reviews for more than 65 clinical questions [25]
Evidence from data	Phase 4: Disseminate, implement, evaluate and update	Evaluation of the 2018 International PCOS Guideline implementation into practice has evaluated alignment of practice with recommendations, as well as alignment of patient and healthcare provider satisfaction with care, to inform ongoing improvement [23], thus informing model of care framework being scaled internationally
Evidence from implementation	Phase 4: Disseminate, implement, evaluate and update	In the 2018 International PCOS evidence-based guidelines, a free accessible patient app, ASKPCOS, was co-designed with patients and has been implemented with more than 37 000 women in 186 countries [24]

Secondly, the LHS can help address issues downstream during the implementation phase (phase 4) of EBG development by providing a whole-of-system framework that removes the traditional silos separating EBG development from implementation and evaluation. Given that guideline consumers, implementers and developers have been involved in EBG development up to this point, the co-design of strategies to implement, disseminate and evaluate can occur in a more seamless manner through embedding recommendations in different mediums appropriate for context. For example, surfacing guidelines through evidence from data by developing digitally structured guidelines that may be integrated into electronic health records [22], utilising changes in data reflecting changes in practice over time due to guideline implementation as a way to evolve models of care [23], and developing patient-centred apps to improve patient education and present recommendations in a way that resonates with healthcare consumers [24].

However, whilst the LHS provides a theoretical framework to better integrate EBG development into the health system as a valuable mechanism to align the priorities of EBM and patient-centred care, practical bottlenecks remain, particularly in ensuring the currency of the systematic reviews that are necessary within EBG development. Clinical questions elicited from stakeholder engagement during phase I of EBG development can amount to more than 60 after priority setting [17], with each clinical question potentially requiring a systematic review in phase 2. This process presents the most time-consuming part of EBG development, with the average systematic review requiring just over a year (67 weeks on average [26]) to complete, and costing US$ 141 194.80 per review [27]. However, with the ongoing pace of research evidence generation, estimates have indicated that 7% of systematic reviews are out of date upon publication, with at least 23% needing to be updated within 2 years [28], thus raising questions surrounding the ongoing sustainability of current, mostly manual methods of evidence synthesis, and ultimately posing systematic risks to the sustainability of current evidence-based guidelines.

## The evidence ecosystem and evidence synthesis automation: addressing temporal bottlenecks in EBG development

Fortunately, the past decade has also heralded the notion of the evidence ecosystem in a bid to better understand such risks, that is, the recognition for the need of a whole-of-system lens to study the drivers and contextual relationships that shape how evidence is generated, synthesized and translated. Within this, an emergent and vibrant research community has emerged to develop new tools and methodologies to address the aforementioned bottlenecks and flaws in evidence synthesis. There has been a distinct shift, particularly over the course of the COVID-19 epidemic, towards more agile, continuously updated forms of evidence [29], with the rise of the living map [30] and living guideline [31] promising to better close the temporal gap between evidence generation and synthesis.

Technological enablers have also emerged to support such forms of evidence. The automation of systematic reviews has now spawned a new subfield of its own, with annual conferences and hackathons [32] that are potent generators of new ideas and tools to better streamline the evidence synthesis processes, with increasing evidence indicating significant savings in effort and cost [33]. Automated title and abstract screening is rapidly maturing, with data extraction an ongoing area of active research [34]. In addition, the rise of mega bibliographic databases such as OpenAlex [35] aggregate academic databases such as PubMed and Scopus and preprint servers such as ArXiV and MedRXiv and now promise centralized, programmatic access to the majority of research evidence, further paving the way for truly automated, living forms of evidence and seamless, almost real-time integration into health system infrastructure.

Collectively, these developments represent an emerging blueprint and growing consensus [36] for the future infrastructure and standards of research evidence synthesis in learning health systems. Concerted efforts, partnerships and standards built on open science principles must now be developed to ensure the continued development and adoption of innovative tools and methods within the evidence ecosystem at large. This is especially required if learning health systems are to fulfil their promise of finally closing the loop on evidence-based practice and practice-based evidence to drive sustained, scalable impact.

## Conclusion

It is evident that much of the social and technological headwinds that accelerated the adoption of EBM in the 1980s are still front and centre with the emergence of the learning health system and evidence ecosystem. Specifically, (i) the ongoing big data revolution and the resurgence of artificial intelligence research, alongside (ii) the urgent need to balance evidence-based rigour at a population scale whilst remaining sensitive to the complex needs of the individual patient. The LHS and evidence ecosystem thus pose complementary approaches in capitalizing on these headwinds towards paving the way for continued evolution and improvement of healthcare in a complex dynamic system.

The LHS offers a way forward for clinical practice that recognizes the validity of many sources of evidence including stakeholders’ priorities. The evidence ecosystem within the LHS offers a vehicle by which the traditional tools of EBM may be configured and improved upon to improve efficiencies and bring evidence generation and synthesis closer to the world of evidence implementation, translation and use. Both seek to consolidate the great advances across fields such as artificial intelligence, evidence-based medicine, person-centred care, implementation science and many other areas to drive health system change. The opportunity now is for leaders and stakeholders to come together and co-deliver the pathways, infrastructure and enablers to operationalise these frameworks and deliver health benefits for the community, as the ultimate funder and beneficiary of both research and healthcare.

## References

[1] Sackett DL, Rosenberg WMC, Gray JAM, Haynes RB, Richardson WS (1996). Evidence based medicine: what it is and what it isn’t. BMJ.

[2] Guyatt G, Cairns J, Churchill D, Cook D, Haynes B, Hirsh J (1992). Evidence-based medicine: a new approach to teaching the practice of medicine. JAMA.

[3] Haynes RB, McKibbon KA, Walker CJ, Ryan N, Fitzgerald D, Ramsden MF (1990). Online access to MEDLINE in clinical settings. A study of use and usefulness. Ann Intern Med.

[4] Haynes RB, McKibbon KA, Walker CJ, Mousseau J, Baker LM, Fitzgerald D (1985). Computer searching of the medical literature. Ann Intern Med.

[5] Clarke M, Chalmers I (2018). Reflections on the history of systematic reviews. BMJ EBM.

[6] Correa VC, Lugo-Agudelo LH, Aguirre-Acevedo DC, Contreras JAP, Borrero AMP, Patiño-Lugo DF (2020). Individual, health system, and contextual barriers and facilitators for the implementation of clinical practice guidelines: a systematic metareview. Health Res Policy Syst.

[7] Fernandez A, Sturmberg J, Lukersmith S, Madden R, Torkfar G, Colagiuri R (2015). Evidence-based medicine: is it a bridge too far?. Health Res Policy Syst.

[8] McDonald S, Turner S, Page MJ, Turner T (2022). Most published systematic reviews of remdesivir for COVID-19 were redundant and lacked currency. J Clin Epidemiol.

[9] Glasziou PP, Sanders S, Hoffmann T (2020). Waste in covid-19 research. BMJ.

[10] Li W, Gurrin LC, Mol BW (2022). Violation of research integrity principles occurs more often than we think. Reprod Biomed Online.

[11] Institute of Medicine Roundtable on Evidence-Based M. In: Olsen L, Aisner D, McGinnis JM, editors. The Learning Healthcare System: Workshop Summary. Washington (DC): National Academies Press (US) Copyright © 2007, National Academy of Sciences; 2007. 21452449

[12] Enticott J, Johnson A, Teede H. Learning health systems using data to drive healthcare improvement and impact: a systematic review. BMC Health Serv Res. 2021;21. https://semanticscholar.org/paper/a01329edd9b3d5716d98b252509d24e2c330bf29. 10.1186/s12913-021-06215-8 PMC7932903 33663508

[13] Geist MR (2010). Using the Delphi method to engage stakeholders: a comparison of two studies. Eval Program Plann.

[14] Archibald MM, Wittmeier K, Gale M, Ricci F, Russell K, Woodgate RL (2021). Living labs for patient engagement and knowledge exchange: an exploratory sequential mixed methods study to develop a living lab in paediatric rehabilitation. BMJ Open.

[15] Blood Z, Tran A, Caleo L, Saw R, Dieng M, Shackleton M (2021). Implementation of patient-reported outcome measures and patient-reported experience measures in melanoma clinical quality registries: a systematic review. BMJ Open.

[16] Cumpston M, Li T, Page MJ, Chandler J, Welch VA, Higgins PTJ, et al. Updated guidance for trusted systematic reviews: a new edition of the Cochrane Handbook for Systematic Reviews of Interventions. http://ovidsp.ovid.com/ovidweb.cgi?T=JS&PAGE=reference&D=coch&NEWS=N&AN=00075320-100000000-11859. Accessed 1 Jan 2019. 10.1002/14651858.ED000142 PMC10284251 31643080

[17] Teede HJ, Misso ML, Costello MF, Dokras A, Laven J, Moran L (2018). Recommendations from the international evidence-based guideline for the assessment and management of polycystic ovary syndrome†‡. Hum Reprod.

[18] Guyatt GH, Oxman AD, Vist GE, Kunz R, Falck-Ytter Y, Alonso-Coello P (2008). GRADE: an emerging consensus on rating quality of evidence and strength of recommendations. BMJ.

[19] Lowes L, Noritz G, Newmeyer A, Embi P, Yin H, Smoyer W. ‘Learn From Every Patient’: implementation and early results of a learning health system. Dev Med Child Neurol. 2017;59. https://semanticscholar.org/paper/17f69d5bc6bb75c4bfa890711ba3db140df841c1. 10.1111/dmcn.13227 27545839

[20] Safaeinili N, Brown-Johnson C, Shaw JG, Mahoney M, Winget M (2020). CFIR simplified: pragmatic application of and adaptations to the Consolidated Framework for Implementation Research (CFIR) for evaluation of a patient-centered care transformation within a learning health system. Learn Health Syst.

[21] Pottie K, Magwood O, Rahman P, Concannon T, Alonso-Coello P, Jaramillo Garcia A (2021). GRADE Concept Paper 1: Validating the ‘F.A.C.E’ instrument using stakeholder perceptions of feasibility, acceptability, cost, and equity in guideline implement. J Clin Epidemiol..

[22] Shah S, Yeheskel A, Hossain A, Kerr J, Young K, Shakik S (2021). The impact of guideline integration into electronic medical records on outcomes for patients with diabetes: a systematic review. Am J Med.

[23] Tay CT, Pirotta S, Teede HJ, Moran LJ, Robinson T, Skouteris H (2021). Polycystic ovary syndrome models of care: a review and qualitative evaluation of a guideline-recommended integrated care. Semin Reprod Med.

[24] Ramasamy VA, Rhonda GM, Boyle JA (2021). A comprehensive PCOS research and guideline translation program to improve practice. Semin Reprod Med..

[25] TECHNICAL REPORT FOR: International evidence‐based guideline for the assessment and management of polycystic ovary syndrome 2018. https://www.monash.edu/__data/assets/pdf_file/0020/1412282/PCOS-Guideline_Technical-report.pdf.

[26] Borah R, Brown AW, Capers PL, Kaiser KA (2017). Analysis of the time and workers needed to conduct systematic reviews of medical interventions using data from the PROSPERO registry. BMJ Open.

[27] Michelson M, Reuter K (2019). The significant cost of systematic reviews and meta-analyses: a call for greater involvement of machine learning to assess the promise of clinical trials. Contemp Clin Trials Commun.

[28] Shojania KG, Sampson M, Ansari MT, Ji J, Doucette S, Moher D (2007). How quickly do systematic reviews go out of date? A survival analysis. Ann Intern Med.

[29] COVID-NMA. The COVID-NMA initiative: a living mapping and living systematic review of Covid-19 trials. 2020. https://covid-nma.com/dataviz/. Accessed 7 Jun 2022.

[30] Miake-Lye IM, Hempel S, Shanman R, Shekelle PG (2016). What is an evidence map? A systematic review of published evidence maps and their definitions, methods, and products. Syst Rev.

[31] Hill K, English C, Campbell BCV, McDonald S, Pattuwage L, Bates P (2022). Feasibility of national living guideline methods: the Australian Stroke Guidelines. J Clin Epidemiol.

[32] Evidence Synthesis Hackathon. 2022. https://www.eshackathon.org/.

[33] Shemilt I, Noel-Storr A, Thomas J, Featherstone R, Mavergames C (2022). Machine learning reduced workload for the Cochrane COVID-19 Study Register: development and evaluation of the Cochrane COVID-19 Study Classifier. Syst Rev.

[34] Marshall IJ, Wallace BC (2019). Toward systematic review automation: a practical guide to using machine learning tools in research synthesis. Syst Rev.

[35] Priem J, Piwowar H, Orr R. OpenAlex: a fully-open index of scholarly works, authors, venues, institutions, and concepts. arXiv; 2022. https://arxiv.org/abs/2205.01833.

[36] Vandvik PO, Brandt L (2020). Future of evidence ecosystem series: evidence ecosystems and learning health systems: why bother?. J Clin Epidemiol.

